# Mental health literacy about schizophrenia and depression: a survey among Chinese caregivers of patients with mental disorder

**DOI:** 10.1186/s12888-017-1245-y

**Published:** 2017-03-09

**Authors:** Shubao Chen, Qiuxia Wu, Chang Qi, Huiqiong Deng, Xuyi Wang, Haoyu He, Jiang Long, Yifan Xiong, Tieqiao Liu

**Affiliations:** 1 Mental Health Institute of the Second Xiangya Hospital, Central South University, The China National Clinical Research Center for Mental Health Disorders, National Technology Institute of Psychiatry, Key Laboratory of Psychiatry and Mental Health of Hunan Province, Changsha, Hunan 410011 China; 2 Department of Psychiatry and Behavioral Sciences, University of Texas Medical School at Houston, Houston, USA

**Keywords:** Mental health literacy, Beliefs, Caregivers, China

## Abstract

**Objective:**

To investigate the knowledge of schizophrenia and depression among caregivers of patients with mental disorder in China.

**Method:**

A convenience sample of 402 caregivers at the Department of Psychiatry of a general hospital in China was investigated (response rate 95.7%), using vignettes based investigation methodology.

**Results:**

The number of caregivers using the term “depression” to describe the depression vignette was 43.6%, which was significantly higher than the number of caregivers using the term “schizophrenia” to describe the schizophrenia one (28.5%). A high percentage of caregivers believed that “psychiatrist”, “psychologist” and “close family members” would be helpful, and the top three most helpful interventions were “becoming more physically active”, “getting out and learning more” and “receiving psychotherapy”. The number of caregivers endorsed “antipsychotics” and “antidepressants” as helpful for the schizophrenia and the depression vignettes were 82.0 and 80.7%, respectively. Regarding the causes of mental illness, items related to psychosocial factors, including “daily problems” and “work or financial problems”, and “weakness of character” were highly rated, with half considered genetic or chemical imbalance causes.

**Conclusion:**

Caregivers expressed a high knowledge about treatments and interventions of mental disorders. But there are still some areas, particularly regarding the recognition and causes of mental disorders, that are in need of improvement. This is particularly the case for schizophrenia.

**Electronic supplementary material:**

The online version of this article (doi:10.1186/s12888-017-1245-y) contains supplementary material, which is available to authorized users.

## Background

Neuropsychiatric disorders are highly prevalent globally, and becoming the most common causes of illnesses and disabilities [1, 2]. The data from the Global Burden of Diseases, Injuries, and Risk Factors Study 2010 (GBD 2010) informed us that mental and substance use disorders were the leading cause of years lived with disability worldwide, with depressive disorders accounting for 40.5% of disability-adjusted life years [3]. Schizophrenia is another highly disabling mental disorders. Bottlender et al. Reported that severe to very severe levels of social disability were found in 64% of schizophrenic patients [4]. Thus, the levels of caregiver burden caring for a relative with schizophrenia, including family finance, family routine, family leisure, family interaction, physical and mental health were high, especially among Chinese families [5–7].

The term mental health literacy (MHL) was introduced by Jorm et al. in 1997 [8]. It refers to knowledge and beliefs about mental disorders, which aid in their recognition, management and prevention. According to Jorm et al., MHL includes the following six components: (1) the ability to recognize specific disorders; (2) knowing how to seek mental health information; (3) knowledge of risk factors and causes; (4) knowledge of self-treatments; (5) knowledge of professional help available; (6) attitudes that promote recognition and appropriate help-seeking. There is an assumption that individuals who have a higher level of MHL will be able to recognize mental disorders earlier and be more willing to seek professional help [9]. According to this assumption, improved MHL is theorized to increase rates of early help-seeking for mental health problems and therefore increases the probability of successful treatment and reduces the chances of the disorder becoming more severe. Thus, improving public’s MHL will finally help reduce the global burden caused by neuropsychiatric disorders.

Since the introduction of the term MHL, a series of studies have been conducted among various populations in developed and developing countries. Jorm et al. conducted pioneer research on MHL in Australia in 1995. They found that the public tend to show a low level of recognition of mental disorders and have a negative view of medical treatments [10]. In 2003 and 2011, they have conducted another two studies respectively [11, 12], and found that public’s recognition of depression was improved over time and respondents showed more positive ratings for a range of interventions. Wang et al. also found that MHL in Shanghai, China appear to be increasing over time [13]. While Yu et al. confirmed that there was much room for improvement with regard to MHL promotion in rural areas of China [14].

The ideographic Chinese writing system expresses the meaning of schizophrenia as “the disease of disorganized mind” [15]. The patients who are suffering from mental health problems have often been called as “*dian zi*” or “*feng zi*” in China. As a collective society like China, people are trying to establish a strong and tough statue in the public, and afraid of losing one’s honor and reputation to others (which is called “losing face”). People perceived mental disorder negative, the Chinese characters directly convey a deeply ingrained stigma. Patients with mental disorders may be reluctant to seek professional help from mental health services in order to protect their families from “losing face” [16, 17]. Only a small proportion of patients have ever seen a mental health professional [1]. Furthermore, it has been shown that lacking of MHL was one of the important factors for delays in help-seeking [16, 18].

The studies on MHL were almost carried out among the general population, only few focused on the caregivers of patients with mental disorders [19]. The caregivers not only provide the basic care such as long-term assistance of housing and financial aid, but also have to monitor the patient’s mental state, supervise the patient to take medicine on time, identify the early signs of illness, provide emotional support, and help patient access services [20]. The caregivers are expected to assume a variety of roles, so it is understandable that their levels of MHL will affect the patient’s mental status and rehabilitation. Since caregivers play an important role for patient with mental disorder, we undertook a survey to assess the level of MHL among Chinese caregivers to study their attitudes toward people with schizophrenia and depression.

## Methods

### Sample

This survey was conducted between January 2014 and December 2014 in the Department of Psychiatry of a general hospital located in Changsha, China, which is the capital city of Hunan province. We recruited a convenience sample of 402 caregivers aged 16 years and older. The caregivers recruited for this research were the caregivers of people with any mental illness. If there were more than one caregivers of a patient, we chose the one who was the main caregiver and volunteered to complete the questionnaire. We excluded the caregivers who were diagnosed with mental illness themselves.

### Survey methods

It was a cross-sectional descriptive study and used the vignette based investigation methodology developed by Jorm et al. [8]. Participants received a vignette of a person with either schizophrenia or depression randomly. The survey was conducted by two graduate students in the department under the supervision of a psychiatry professor. The questionnaire was administered as a self-completion one and delivered by person, so that each caregiver completed independently. It took participants 20–30 min to complete the questionnaire. If a caregiver had difficulty reading the questionnaire due to poor sight or low literacy, investigators read the items verbally to the participant and recorded the responses.

### Instrument

The instrument used for data collection was translated and modified by Dr. Liu Wei after obtaining the permission of the original author, Anthony Jorm. This questionnaire has previously been used to investigate levels of MHL among a selected group of psychiatrists and registered nurses in China. We used the same vignettes as those used in the Liu et al. research. Details regarding the procedures used to translate and modify the questionnaire to simplified Chinese, establish its reliability and validity can be found in Liu et al. [21]. In the current study, participants responded to vignettes identical to the two developed by Jorm et al. [8] to meet the International Classification of Disease-10 and the Diagnostic and Statistical Manual-IV criteria for depression and schizophrenia (see Additional file 1). The questionnaire was presented in Chinese.

The demographic information of the participants was collected first, followed by a schizophrenia vignette or a depression vignette. Following the vignette, the caregivers were asked to select one “most likely” diagnosis related to the vignette from a series of diagnostic options. After that, they were asked to rate the likely effect of a range of interventions (rated as “helpful”, “harmful” or “neither”) to assess their beliefs about the treatments [8, 10]. About their knowledge of cause and risk factors of mental disorders, respondents were asked to rate the likelihood of a range of possible causes including “viruses”, “allergic reactions”, “daily problems” and so on. The options rated as “very likely”, “likely”, “not likely”, “depends” or “don’t know” [22].

### Statistical analyses

All statistical analyses were conducted in SPSS, version 18.0. We performed descriptive analyses for the percentage of each endorsed statement. After that, we conducted chi-square calculations (*χ* ^2^ tests) for categorical variables to compare the recognition and intervention beliefs toward the person described in the two vignettes. Statistical significance was set at *p* < 0.05.

## Results

We distributed 420 questionnaires, and a total of 402 caregivers completed the questionnaires with a response rate of 95.7% (402/420). As shown in Table 1, 58.5% were female; 36.8% were between 36 to 45 years of age, the mean age was 41.1 (SD: 10.4); 88.1% were married; 33.1% had 13 or more years of education; 28.9% were patient’s mother.

**Table 1:** Demographic characteristics of caregivers

Variables	Number	Percent
Gender		
Male	167	41.5
Female	235	58.5
Age (years)		
16–25	44	10.9
26–35	70	17.4
36–45	148	36.8
46–55	108	26.9
56–65	29	7.2
≥66	3	0.7
Marital status		
Currently married	354	88.1
Not married	48	11.9
Education (years)		
0–6	25	6.2
7–9	135	33.6
9–12	109	27.1
≥13	133	33.1
Relationship to the patient		
Father	78	19.4
Mother	116	28.9
Sibling	48	11.9
Other relationship^a^	160	39.8

^a^Other relationship including patients’ intimate partner, friend, children and so on

### Recognition of mental disorder

As shown in Table 2, the term “schizophrenia” was used by 28.5% (*n* = 57) of caregivers for the schizophrenia vignette, with 43.6% (*n* = 88) used the term “depression” to describe the depression vignette. This difference in recognition rates was statistically significant (*χ* ^2^ = 9.890, df = 1, *p* = 0.002). For the schizophrenia vignette, 23.5% of the caregivers nominated the vignette as “depression”. While 14.4 and 10.9% of the caregivers nominated the depression vignette as “psychological problems” and “nervous breakdown”, respectively.

**Table 2:** Caregivers’ rating of each category to describe the problem shown in the vignette

Category nominated	Schizophrenia (*N* = 200)		Depression (*N* = 202)	
	N	%	N	%
Nervous breakdown	8	4.0	22	10.9
Depression	47	23.5	88	43.6
Schizophrenia	57	28.5	9	4.5
Psychological problems	14	7.0	29	14.4
Mental illness	44	22.0	21	10.4
Physical illness	0	0	2	1.0
Emotional problems	3	1.5	5	2.5
Self-esteem	1	0.5	0	0
Spirit intrusion	0	0	0	0
Phobia	11	5.5	1	0.5
Social phobia	6	3.0	3	1.5
GAD	2	1.0	14	6.9
PTSD	0	0	0	0
Acute anxiety attack	3	1.5	5	2.5
OCD	3	1.5	1	0.5
Personality disorder	0	0	0	0
Caused by physical illness	0	0	2	1.0
Other	0	0	0	0
Don’t know	1	0.5	0	0

*GAD* generalized anxiety disorder, *PTSD* post-traumatic stress disorder, *OCD* obsessive-compulsive disorder

### Beliefs about treatments and other interventions

A descriptive analysis about people, medications and other interventions that may be helpful or harmful to the person described in the vignette was conducted. As showed in Table 3, for the schizophrenia vignette, “psychiatrist” was considered as the most helpful people, followed by “psychologist” and “close family members”. “Psychologist” was considered as the most helpful people for the depression vignette, followed by “psychiatrist” and “close family members”. There were 69.0 and 62.9% of caregivers thought “dealing with problem on his/her own” would be harmful for the schizophrenia and the depression vignettes separately, with 61.0 and 54.5% considered “religious leader” as harmful, respectively. The top three rated helpful interventions (above 90%) were “becoming more physically active”, “getting out and learning more” and “receiving psychotherapy” for the two vignettes; while “having occasional drink”, “electroconvulsive therapy (ECT)” and “being kept at home” were among the most harmful interventions.

**Table 3:** Caregivers’ rating of perceived helpfulness/harmfulness for people and interventions

Method of help	Schizophrenia (*N* = 200)		Depression (*N* = 202)	
	Helpfulness	Harmfulness	Helpfulness	Harmfulness
	*n* (%)	*n* (%)	*n* (%)	*n* (%)
People who could help				
GP or family doctor	125 (62.5)	5 (2.5)	140 (69.3)	6 (3.0)
Pharmacist	81 (40.5)	25 (12.5)	81 (40.1)	29 (14.4)
Counselor	148 (74.0)	7 (3.5)	144 (71.3)	5 (2.5)
Social worker	92 (46.0)	18 (9.0)	92 (45.5)	12 (5.9)
Telephone counseling service	92 (46.0)	14 (7.0)	100 (49.5)	15 (7.4)
Psychiatrist	194 (97.0)*	2 (1.0)	184 (91.1)	1 (0.5)
Mental health nurse	178 (89.0)**	1 (0.5)	157 (77.7)	2 (1.0)
Psychologist	192 (96.0)	8 (4.0)	188 (93.1)	0 (0)
Close family members	185 (92.5)	2 (1.0)	177 (87.6)	1 (0.5)
Close friends	175 (87.5)	3 (1.5)	167 (82.7)	3 (1.5)
Traditional healer/Chinese medicine doctor	105 (52.5)	15 (7.5)	103 (51.0)	12 (5.9)
Dealing with problem on his/her own	23 (11.5)*	138 (69.0)	42 (20.8)	127 (62.9)
Religious leader	14 (7.0)	122 (61.0)	15 (7.4)	110 (54.5)
Interventions				
Becoming more physically active	189 (94.5)	4 (2.0)	194 (96.0)	1 (0.5)
Reading about the problem	128 (64.0)	6 (3.0)	144 (71.3)	8 (4.0)
Getting out and learning more	186 (93.0)	1 (0.5)	182 (90.1)	2 (1.0)
Being kept at home	36 (18.0)**	87 (43.5)	61 (30.2)	62 (30.7)
Attending courses on relaxation	153 (76.5)	9 (4.5)	164 (81.2)	7 (3.5)
Massage and having a rest	139 (69.5)**	5 (2.5)	167 (82.7)	1 (0.5)
Cutting out alcohol	166 (83.0)	10 (5.0)	161 (79.7)	6 (3.0)
Having occasional drink	23 (11.5)**	112 (56.0)	45 (22.3)	108 (53.5)
Practicing Chi Kung/Tai Chi therapy	85 (42.5)	18 (9.0)	102 (50.5)	32 (15.8)
Receiving acupuncture	70 (35.0)	23 (11.5)	83 (41.1)	22 (10.9)
Receiving psychotherapy	188 (94.0)	1 (0.5)	189 (93.6)	0 (0)
Participating in hypnosis	112 (56.0)	20 (10.0)	132 (65.3)	16 (7.9)
Receiving aromatherapy	53 (26.5)	41 (20.5)	68 (33.7)	22 (10.9)
Being admitted to a psychiatric ward in a general hospital	162 (81.0)**	8 (4.0)	138 (68.3)	26 (12.9)
Being admitted to a psychiatric hospital	140 (70.0)**	28 (12.0)	104 (51.5)	61 (30.2)
Receiving electroconvulsive therapy (ECT)	51 (25.5)	95 (47.5)	43 (21.3)	96 (47.5)
Going on a special diet	91 (45.5)	19 (9.5)	100 (49.5)	15 (7.4)

*GP* general practitioner. *Difference between two vignettes *p* < 0.05. **Difference between two vignettes *p* < 0.01

Compared to the depression vignette, the rates of caregivers considered “psychiatrist” (*χ* ^2^ = 6.255, df = 1, *p* = 0.012), “mental health nurse” (*χ* ^2^ = 9.202, df = 1, *p* = 0.002), “being admitted to a psychiatric ward in a general hospital” (*χ* ^2^ = 8.538, df = 1, *p* = 0.003) and “being admitted to a psychiatric hospital” (*χ* ^2^ = 14.441, df = 1, *p* < 0.001) as helpful were significantly higher for the schizophrenia vignette. The helpful rates of “dealing with problem on his/her own” (*χ* ^2^ = 6.402, df = 1, *p* = 0.011), “being kept at home” (*χ* ^2^ = 8.168, df = 1, *p* = 0.004), “massage and having a rest” (*χ* ^2^ = 9.594, df = 1, *p* = 0.002) and “having occasional drink” (*χ* ^2^ = 8.306, df = 1, *p* = 0.004) were lower for the schizophrenia vignette than for the depression one.

As showed in Table 4, caregivers considered “antipsychotics” (82.0%) and “antidepressants” (80.7%) as the most helpful medications for the schizophrenia and the depression vignettes, respectively. “Vitamins and minerals” were also relatively highly rated compared to other medications, with 40.5% (for the schizophrenia vignette) and 47.0% (for the depression vignette) of caregivers rating these as helpful, respectively. “Sleeping pills” was considered as harmful by 47.5% (for the schizophrenia vignette) and 35.6% (for the depression vignette) of caregivers. The rate of caregivers endorsed “sleeping pills” as helpful was higher for the depression vignette (*χ* ^2^ = 7.400, df = 1, *p* = 0.007), and the helpful rate of “antipsychotics” was significantly higher for the schizophrenia one (*χ* ^2^ = 21.926, df = 1, *p* < 0.001).

**Table 4:** Caregivers’ rating of perceived helpfulness/harmfulness for medications

Medications	Schizophrenia (*n* = 200)		Depression (*n* = 202)	
	Helpfulness	Harmfulness	Helpfulness	Harmfulness
	n (%)	n (%)	n (%)	n (%)
Vitamins and minerals	81 (40.5)	14 (7.0)	95 (47.0)	15 (7.4)
Laxatives	28 (14.0)	80 (40.0)	22 (10.9)	105 (52.0)
Herbal medicines	72 (36.0)	32 (16.0)	75 (37.1)	29 (14.4)
Pain relievers	22 (11.0)	113 (56.5)	30 (14.9)	111 (55.0)
Antidepressants	157 (78.5)	18 (9.0)	163 (80.7)	20 (9.9)
Antibiotics	34 (17.0)	76 (38.0)	33 (16.3)	91 (45.0)
Sleeping pills	60 (30.0)**	95 (47.5)	87 (43.1)	72 (35.6)
Antipsychotics	164 (82.0)**	20 (10.0)	123 (60.9)	44 (21.8)
Tranquillizers	104 (52.0)	46 (23.0)	103 (51.0)	50 (24.8)
Anti-anxiety drugs	154 (77.0)	15 (7.5)	156 (77.2)	19 (9.4)

*Difference between two vignettes *p* < 0.05; **Difference between two vignettes *p* < 0.01

### Knowledge of causes and risk factors

As showed in Table 5, caregivers considered that psychosocial factors including “daily problems”, “work related or financial problems” as the most common likely causes for schizophrenia and depression (79 ~ 82%). “Weakness of character” was considered as causes and risk factors by 72.5% (for the schizophrenia vignette) and 68.3% (for the depression vignette) of caregivers. And approximately half of the caregivers believed schizophrenia and depression were “inherited or genetic”, or caused by “chemical imbalance in the brain”. The rate of caregivers considered “death of someone close” as causes and risk factors for the depression vignette was higher than for the schizophrenia one (*χ* ^2^ = 11.724, df = 1, p = 0.001).

**Table 5:** Caregivers’ rating of causes and risk factors as “very likely” and “likely”

Causes and risk factors	Schizophrenia (*n* = 200)		Depression (*n* = 202)	
	*n*	(%)	*n*	(%)
Virus of infection	30	(15.0)	32	(15.8)
Allergy	35	(17.5)	45	(22.3)
Daily problems	163	(81.5)	160	(79.2)
Work or financial problems	164	(82.0)	162	(80.2)
Death of someone close**	100	(50.0)	135	(66.8)
Recently traumatic event	129	(64.5)	146	(72.3)
Problems from childhood	102	(51.0)	104	(51.5)
Inherited or genetic	96	(48.0)	103	(51.0)
Chemical imbalance in the brain	86	(43.0)	105	(52.0)
Nervous person	119	(59.5)	123	(60.9)
Weakness of character	145	(72.5)	138	(68.3)

*Difference between two vignettes *p* < 0.05; **Difference between two vignettes *p* < 0.01

## Discussion

This study was conducted to investigate MHL of schizophrenia and depression among caregivers of patient with mental disorder in China. As caregivers of patient with mental illness, their knowledge of MHL is expected to be better than others. However, in this survey, we found that the rates of recognition for the schizophrenia vignette was relatively low, with only 28.5% of caregivers used the term “schizophrenia”. This rate was similar to the value of 37.3% found in the survey conducted among general population in Australia in 2011 [23]. There is evidence that recognition of mental disorders is essential for facilitating early help-seeking [24]. Low recognition of schizophrenia may lead to delayed help-seeking and worse the situation. Efforts should be taken to increase caregivers awareness of schizophrenia.

Caregivers demonstrated a moderate rate of recognition for the depression vignette (43.6%), which was significantly higher than the schizophrenia vignette in this survey (*χ* ^2^ = 9.890, df = 1, *p* = 0.002). It is partly because the government and the media have put much greater emphasis on depression, due to its greater potential to commit suicide. This may also explain the highly use of the term “depression” for the schizophrenia vignette, and arise as a result that “depression” become a more acceptable term and are commonly used across mental disorders [11]. But whether the difference of recognition between the two vignettes was due to caregivers’ lack of MHL, or their defense mechanism was still uncertain, and needed continued exploration.

When asked about the interventions, caregivers tended to endorse the helpfulness of professional help from “psychiatrist”, “mental health nurse”, “psychologist” and “receiving psychotherapy”. It was encouraging to note that caregivers would like to seek professional help for patients. Also, the self-help interventions such as help from “close family members” and “close friends”, “becoming more physically active” and “getting out and learning more” were also highly rated (above 82%) for the two vignettes. There is evidence that such self-help interventions are helpful for those with schizophrenia and depression [25]. The high endorsements indicate a more holistic, recovery-orientated framing of what is important in the treatment. Although this does not treat the psychiatric condition, it is necessary for the holistic treatment of people with the condition. But, it has to be pointed out that first seek help from these self-help interventions instead of professional one may not be effective and it is possible that such beliefs may delay access to appropriate treatments [11]. So equipped caregivers the knowledge of when to seek professional help and self-help methods seem to be necessary.

“General practitioner (GP) or family doctor” and “counselor” were highly rated as helpful for both vignettes. Chinese government try to build community general practitioner (GP) as the first contact for health problem in order to improve the health of a huge population [26]. But their interest in psychiatric treatment at the moment is not necessarily [26, 27]. It is difficult to say that their ability to recognize and treat psychiatric patients is sufficient. Also, there is still no standardized training for counselors in China. In order to make sure that GPs and counselors could offer appropriate and prompt referrals, it is essential to set up a sophisticated GP and counselor medical system. On the other hand, GPs and counselors should equip themselves both with the skills necessary to conduct basic psychiatric assessments and the knowledge of MHL and services available in the community [28].

There were about half of the caregivers perceived traditional healer/Chinese medicine doctor and Chi Kung/Tai Chi therapy as helpful. In China, it is common to use both Western and traditional Chinese medicine for certain diseases [9]. According to the statements of the caregivers, some of the patients had tried traditional Chinese medicine before they first came to the clinic. However, evidence showed that traditional treatments might not be helpful for mental disorders, but could also lead to a delay in seeking medical professional help [29]. Equipped caregivers with the function of traditional Chinese medicine and other traditional treatments should be taken into account in future health education. Furthermore, provide traditional Chinese medical doctors with knowledge of mental health literacy, teach them how to refer patients and inform them the location of mental health services are essential.

As caregivers of mental disorders, they have got through worse untreated situation with the patients. This can explain the result that most of them endorsed dealing with it alone as harmful. This endorsement rate was close to that of the public belief of Australia [24]. Caregivers’ views on electroconvulsive therapy (ECT) were quite negative, as there were 47.5% endorsed ECT as harmful for the two vignettes. ECT is utilized worldwide as one of the most effective biological treatment for various severe, treatment-resistant psychiatric disorders, particularly, for major depressive disorder (MDD) and schizophrenia [30]. Caregivers’ negative views on ECT might lead to the unwillingness to choose this biological treatment when necessary. Well inform patients and caregivers of the effect of ECT treatment is important.

There were notably more caregivers rating “psychiatrist” and “mental health nurse” as helpful for the schizophrenia vignette than the depression one. Individuals suffering from schizophrenia sometimes behave erratically, such as whispering or talking to themselves. They have been characterized as having a “split personality” and inaccurately depicted as violent by the social media [31]. It was not surprising that a higher percentage of caregivers endorsed psychiatrist and mental health nurse as helpful for schizophrenia. This was consistent with the result that a significantly higher rate of caregivers considered inpatient treatments, including “being admitted to a psychiatric ward in a general hospital” and “to a psychiatric hospital”, as helpful for the schizophrenia vignette.

It was a promising result that 82.0 and 80.7% of caregivers considered “antipsychotics” and “antidepressants” as helpful for the schizophrenia and the depression vignettes, respectively. This is mainly because the survey was conducted in a department of psychiatry, caregivers have been told the necessity to take psychiatric medication. Evidence showed that people with an affected relative or friend who had received treatment were more likely to believe in the helpfulness of psychiatric medication [32]. However, it has to be pointed out that above 40% endorsed “vitamins and minerals” as helpful. It should be taken into consideration to help caregivers better understand the medication in further health education.

Phillips et al. reported that more than 84% of caregivers attributed the causes of schizophrenia to social, interpersonal and psychological problems, few accounted to biological and spiritual causes [33]. Consistently, we found that nearly 80% of the caregivers attributed the causes of schizophrenia and depression to psychosocial problems, including “daily problems” and “work or financial problems”. Evidence showed that belief in a weak or nervous personality as a cause of mental illness was associated with increased stigma [34]. The high causal belief of “weakness of character” in this survey may indicate the stigma attitude towards mental disorder. Half of the caregivers considered “genetic factors” and “chemical imbalance in the brain” as likely causes. A recent meta-analysis showed that, beliefs in neurochemical and general biogenetic causation are associated with social distance for schizophrenia, they are also associated with increased belief in dangerousness for all mental disorders [35]. Overall, mental disorder is a complex interaction between biogenetic and psychosocial factors, future studies to understand whether there might be harm in emphasising biological causes of mental disorder due to an increase in stigma is needed. Public health education should acknowledge caregivers the message of multi-factorial aetiology including bio-psycho-social factors [36].

The study has some limitations that must be acknowledged. The main limitation is the selected nature of the sample. Participants in the survey were caregivers who were already in contact with specialist mental health services. Another limitation is the small sample size. The selected and small sample may not represent the average MHL level of caregivers in China. A third limitation is that we do not add questions about the diagnostic labels and treatments that have been given to the caregivers’ relatives, so we don’t know whether the caregivers’ MHL is relevant to their relative’s mental disorder. A fourth limitation is that the response categories were pre-specified. Respondents may not have too much choice rather than the listed one. The cross-sectional design of the study is another limitation, and we are not able to evaluate how the caregivers’ MHL levels influence the patients’ rehabilitation. Additional investigation in more caregivers at different education and location are necessary. Carry out a longitudinal study can be taken into consideration to know better about the changes of MHL in China.

## Conclusions

In conclusion, caregivers in Hunan province expressed a high knowledge about treatments and interventions of mental disorders. But there are still some areas, particularly regarding the recognition and causes of mental disorders, that are in need of improvement. This is particularly the case for schizophrenia. Caregivers showed low recognition of schizophrenia and moderate of depression, endorsed a range of interventions, including professional help and self-help, and contributed causes of mental disorders mainly to psychosocial problems and personal weakness. More intervention programs to increase MHL among Chinese caregivers are necessary, especially educational intervention, and the above aspects should be taken into consideration.

## Additional file

Additional file 1: Provide the details about the characteristics of the hypothetical people in the schizophrenia vignette and the depression vignette in English. (DOCX 12 kb)

## Abbreviations

ECT: Electroconvulsive therapy
GAD: Generalized anxiety disorder
MDD: Major depressive disorder
MHL: Mental health literacy
OCD: Obsessive-compulsive disorder
PTSD: Post-traumatic stress disorder
