# Supplementary material for: Mental health literacy about schizophrenia and depression: a survey among Chinese caregivers of patients with mental disorder
Source: BMC Psychiatry. 2017 Mar 9;17:89. doi: 10.1186/s12888-017-1245-y (PMC5343538; doi:10.1186/s12888-017-1245-y)
Supplement: Additional file 1: — Provide the details about the characteristics of the hypothetical people in the schizophrenia vignette and the depression vignette in English. (DOCX 12 kb) [file 12888_2017_1245_MOESM1_ESM.docx]

**Additional file 1**

**Schizophrenia Vignette**

Zhangsan is 24 and lives at home with his parents. He has had a few temporary jobs since finishing school but is now unemployed. Over the last 6 months he has stopped seeing his friends, and has begun locking himself in his bedroom and refusing to eat with the family or to have a bath. His parents also hear him walking about in his bedroom at night while they are in bed. Even though they know he is alone,they have heard him shouting and arguing as if someone else is there. When they try to encourage him to do more things, he whispers that he won’t leave home because he is being spied upon by the neighbor. They realise he is not taking drugs because he never sees anyone or goes anywhere.

**Depression Vignette**

Lisi is 30 years old. She has been feeling unusually sad and miserable for the last few weeks. Even though she is tired all the time, she has trouble sleeping nearly every night. Mary doesn’t feel like eating and has lost weight. She can’t keep her mind on her work and puts off making any decisions. Even day-to-day tasks seem too much for her. This has come to the attention of Mary’s boss who is concerned about her lowered productivity.
